# Protein Complex Organization Imposes Constraints on Proteome Dysregulation in Cancer

**DOI:** 10.3389/fbinf.2021.723482

**Published:** 2021-08-30

**Authors:** Gökçe Senger, Martin H. Schaefer

**Affiliations:** Department of Experimental Oncology, IEO, European Institute of Oncology IRCCS, Milan, Italy

**Keywords:** protein-protein interactions, cancer, protein complexes, abundance dysregulation, stoichiometry

## Abstract

Protein assembly is a highly dynamic process and proteins can interact in different ways and stoichiometries within a complex. The importance of maintaining protein stoichiometry for complex function and avoiding aggregation of orphan subunits has been demonstrated. However, how exactly the organization of proteins into complexes constrains differential protein abundance in extreme cellular conditions like cancer, where a lot of protein abundance changes occur, has not been systematically investigated. To study this, we collected proteomic data made available by the Clinical Proteomic Tumor Analysis Consortium (CPTAC) to quantify proteomic changes during carcinogenesis and systematically tested five interaction types in complexes to investigate which of these features impact on protein abundance correlation patterns in cancer. We found that higher than expected fraction of protein complex subunits does not show changes in their abundances compared to those in the normal samples. Furthermore, we found that the way proteins interact in complexes indeed constrains their co-abundance patterns. Our results highlight the role of the interactions between the proteins and the need of cancer cells to deal with aberrant changes in protein abundance.

## Introduction

Cancer is a system that is characterized by a large number of somatic molecular alterations. Many of these alterations appear on the level of the transcriptome and proteome of tumor cells. Among other factors, copy number alterations (CNAs), gain or losses in DNA copies, can cause these imbalances in mRNA expression and protein abundances (Mitelman et al., 2007; Beroukhim et al., 2010). However, CNAs are not linearly translated into transcriptome and proteome levels (Liu et al., 2016). Indeed, multi-omics studies estimated that nearly 40% genes are under compensatory post-transcriptional control in response to CNAs (Sousa et al., 2019). Interestingly, those buffered proteins are significantly enriched in protein complexes (Stingele et al., 2012; Gonçalves et al., 2017; Ishikawa et al., 2017; Ryan et al., 2017). Furthermore, it has been shown that members of the same complex have strong co-regulation in protein abundances (Dephoure et al., 2014; Kustatscher et al., 2017; Sousa et al., 2019). Together these results suggest that protein-protein interactions (PPIs) and their organization into complexes limit the dysregulation of protein abundance; however, to which degree complex organization is important and how this buffering is achieved is poorly understood.

Proteins are interacting with each other as complexes to govern cellular functions. Protein complexes are not static units but assemble and disassemble dynamically. This process relies on cellular abundances and localization of proteins, and binding affinities between proteins (Nooren and Thornton, 2003a). For example, quantitative proteomics analysis on different cell lines has shown that stable (core) complex subunits of the anaphase promoting complex/cyclosome (APC/C) are associated with higher cellular abundances and unique stoichiometries while the KIAA1430 subunit, transient interactor of the APC/C, can bind with different stoichiometric ratios (Hein et al., 2015). Furthermore, it has been demonstrated that protein stoichiometries vary depending on the environment such as different tissues and cell types (Ori et al., 2016; Nusinow et al., 2020), which highlights the dynamic organization of proteins in a context-specific manner. Since the pioneering work of Jones and Thornton (Jones and Thornton, 1996), many studies have focused on formalizing PPIs based on binding affinity, composition and stability of the complex, resulting in three main classes; homo- and hetero-oligomeric interactions, non-obligate and obligate interactions, and transient and permanent interactions (Mintseris and Weng, 2003; Nooren and Thornton, 2003a; Acuner Ozbabacan et al., 2011). Moreover, some studies have focused on indirect classification in which functional associations, co-expression, and genetic interactions are considered rather than physical interactions (Eisenberg et al., 2000; Chatr-Aryamontri et al., 2013; Franceschini et al., 2013). However, we still lack a consistent formalization of these partly redundant definitions of interaction types within complexes.

Here, we performed two related tasks: 1) developing a definition and systematic characterization of protein interaction types in complexes; 2) asking how those different interaction types result in (co-)abundance rules between the complex subunits in particular in cellular conditions like cancer that are characterized by dysregulation of gene expression. To answer these questions, we first systematically categorized protein interactions by using structural data, proteomics measurements in different cellular conditions, interaction data, and literature information and then, with the integration of cancer proteomics data, tested how each category impacts on protein abundance correlation patterns in cancer. The outcomes emphasize the role of the interactions between the proteins and their role in complexes in constraining differential protein abundances in cancer.

## Materials and Methods

Code for data analysis is provided as part of the PPIs_Data-Code repository and can be found at https://github.com/SengerG/PPIs_Data-Code.

### Data Processing

Proteomics data used in this publication were generated by the Clinical Proteomic Tumor Analysis Consortium (NCI/NIH). TMT-based log-transformed proteomics data for the following CPTAC cohorts (for which proteomic profiling was done for tumor and normal adjacent tissue samples and processed data is available) were obtained: Colon (COAD) from Vasaikar et al. (2019) (8,067 proteins for 96 tumor and 96 matched normal samples), HBV-related hepatocellular carcinoma (HCC) from Gao et al. (2019) (6,478 proteins for 159 tumor and 159 matched normal samples) and lung (LUAD) from Gillette et al. (2020) (10,699 proteins for 110 tumors and 101 matched normal samples). For the ones for which the confirmatory or discovery study is available, only the representative study was considered. For the COAD cohort, proteomics data were further filtered by excluding proteins that were quantified in less than 50% of the samples, leaving a total number of 6,554 proteins. For the LUAD cohort, proteins mapped to the same gene symbol were merged by taking the mean of the log-transformed TMT values, leaving a total number of 10,316 proteins.

Proteomics data for the available The Cancer Genome Atlas (TCGA) projects were directly obtained from the CPTAC consortium, comprising 3 cohorts, spectral counts for colorectal (COREAD) (Cancer Genome Atlas Network, 2012a; Zhang et al., 2014), and relative abundances for ovarian (OV) (Cancer Genome Atlas Network, 2011; Zhang et al., 2016), and breast (BRCA) (Cancer Genome Atlas Network, 2012b; Mertins et al., 2016). Spectral counts for TCGA COREAD tumor samples were normalized by quantile normalization followed by log-transformation. For the replicated samples, the mean value was considered. This left us 5,561 proteins and 90 samples for COREAD, 7,169 proteins and 174 samples for OV, and 10,625 proteins and 105 samples for BRCA.

### Statistical Analysis

Differential protein abundance analysis was performed by using Wilcoxon test to detect abundance changes between the tumor and matched normal samples and then *p*-values were subjected to multiple testing correction by using the Bonferroni correction. Log2 fold change (log2FC) for proteins was calculated as the median difference of log2 transformed TMT-values between tumor and normal samples. Proteins with absolute log2FC greater than 1 and adjusted *p*-value less than or equal to 0.05 were considered differentially abundant. Associations between the differentially abundant proteins and protein complex subunits, obtained from the CORUM database (Giurgiu et al., 2019), were tested by chi-square test. To create an abundance-matched background set of the same size, we binned proteins based on their abundance and replaced each complex subunit with a non-complex protein from the same bin. Standard deviations in protein abundances were calculated across tumor and normal samples separately and then compared by using *t*-test.

Protein abundance correlations, for all possible protein pairs among proteins covered by proteomics data, were calculated across tumor samples by using the Spearman method separately for all 6 CPTAC cohorts (COAD, HCC, LUAD, COREAD, OV, and BRCA). The difference between the distributions of correlations between members of the same complex and members of different complexes was tested by Wilcoxon test. To this end, correlations from different cohorts for each protein pair were pooled.

### Calculation of Stoichiometric Ratio

Stoichiometry information for proteins was retrieved from the structural data available in the Protein Data Bank (PDB) (for 9,840 PDB entries) (Berman et al., 2000) in March 2020. Uniprot IDs were then converted to gene names and only the human complexes were considered for further analysis, which left us with 8,388 protein complexes comprising 3,075 proteins. For proteins within the same complex, the stoichiometric ratio was calculated as the number of chains of proteins relative to each other. Then, protein pairs were grouped as the ones involved in complexes with even (e.g., 1:1, 2:2, 4:4) and with uneven (e.g., 2:1, 1:2, 3:1) stoichiometric ratio (Supplementary Table S1). Protein pairs that participate in complexes sometimes with even and sometimes with uneven ratio were not included for further analyses.

### Calculation of Co-occurrence Frequency

The known human protein complexes (*n* = 2,916) and their subunits (*n* = 3,664 unique proteins) were obtained from the CORUM database (Giurgiu et al., 2019) (Corum 3.0 current release, September 2018). For each protein pair found together in at least one protein complex, we first counted the number of complexes in which the two proteins were found together. To address a possible bias due to different tendencies of proteins to participate in complexes, we calculated the Jaccard index as a representative of the co-occurrence frequencies by dividing the number of complexes a protein pair is found together by the number of complexes in which at least one of them is found.

### Defining Context-Specific and General Interactions

167,374 protein interactions were obtained from BioPlex Interactome (Huttlin et al., 2021) for the 293T and HCT116 human cell lines. Protein interactions that were detected by the baits targeted in both cell lines were considered, leaving a total number of 33,739 interactions detected in both cell lines (general interactions), and 89,330 interactions detected either in 293T or in HCT116 cell lines (context-specific interactions).

### Defining Competitive and Cooperative Interactions

Protein interaction interface information was extracted from Interactome INSIDER (where interface residues are defined as the ones with a decrease in solvent-accessible surface area equal to or larger than 1.0 Å^2^ upon binding) (Meyer et al., 2018) covering 121,575 experimentally determined human binary interactions among 14,380 proteins. Uniprot IDs were converted to gene names. The binary interactions that have binding site information only for one protein were filtered out, which left us with 70,355 binary protein interactions. To determine if two proteins bind to their common partner in a competitive or cooperative manner, we calculated the Jaccard index (Eq. 1) as a measure of binding similarity between their corresponding interaction sites on the common partner. For the protein pairs that have more than one common partner, the one with the highest Jaccard index was considered for further analyses. Two proteins are classified as competitive if the Jaccard index is equal to or higher than 0.1 and cooperative otherwise.

$$Jaccard\;Index\;=\;\frac{Number\;of\;residues\;in\;A\cap B}{Number\;of\;residues\;in\;A\cup B}$$ (1)

where A and B represent the corresponding interaction sites on the common partner for protein A and B.

### Transient and Permanent Interactions

Transient and permanent PPIs were obtained from Block et al. (2006) (147 permanent and 198 transient interactions among 340 PDB complexes) and from Mintseris and Weng (2003) (209 transient interactions among 207 PDB complexes). After selecting for heterodimers and human complexes and removing the duplicated interactions predicted by both studies, 58 transient and 9 permanent interactions were left.

### Statistical Analysis on Protein-Protein Interaction Types

For each comparison between groups in each class of PPIs, Wilcoxon test was applied.

For each protein pair covered by the structural data obtained from the PDB (Berman et al., 2000), we tested the relationship between proteins by using a linear regression model. In the model, the dependent variable was the protein abundances of the protein with smaller copy number across tumor samples. For the protein pairs with even stoichiometric ratio, the first protein was considered as the dependent variable. Only the relationships where the coefficient of the dependent variable was significantly different from zero (*p*-value < 0.05, linear regression model) were considered for the comparison of slopes between protein pairs with even and uneven stoichiometric ratio. Wilcoxon test was used for the comparison.

## Results

### Complex Members Are to a Certain Degree Protected From Abundance Changes

**FIGURE 1:**
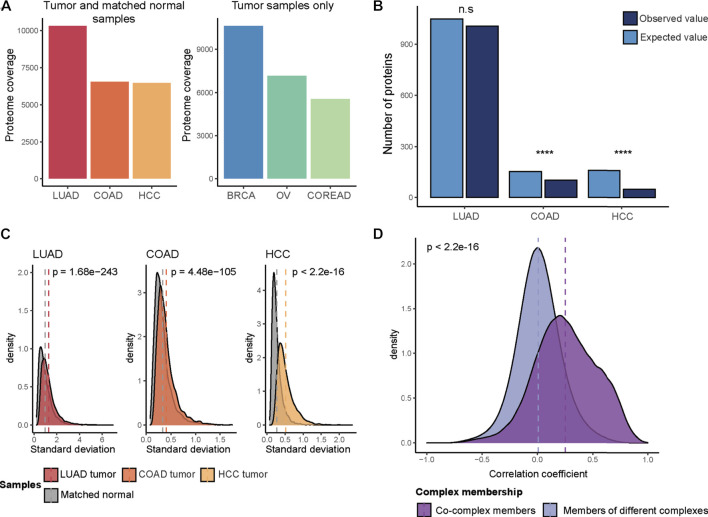
Protein abundance changes in cancer. **(A)** The total number of quantified proteins in each CPTAC cohort after filtering (LUAD, COAD, HCC, BRCA, OV, and COREAD). **(B)** The expected and observed number of differentially abundant proteins in complex subunits. Chi-square test was used to determine if the difference between expected and observed values is statistically significant (n.s: no statistical significance; ****: *p* < 0.0001). **(C)** The difference between the distribution of standard deviations of protein abundances in tumor and matched normal samples. Statistical significance was tested by *t*-test. **(D)** Spearman correlation coefficients of protein pairs across tumor samples. Pairwise correlations were calculated across tumor samples, separately in each cohort (LUAD, COAD, HCC, BRCA, OV, and COREAD) and then pooled. Wilcoxon test was used to test if two distributions are significantly different.

To catalog a representative set of protein abundance changes during carcinogenesis, we obtained proteome quantification data for tumor and normal adjacent tissue samples from the CPTAC consortium, comprising three cohorts; COAD (Vasaikar et al., 2019), HCC (Gao et al., 2019) and LUAD (Gillette et al., 2020) (Figure 1A). Then, differentially abundant proteins were detected between tumor and normal samples. We found a variation in the fraction of quantified proteins that showed significant abundant changes (adjusted *p*-value ≤ 0.05, Wilcoxon test, and absolute log2FC > 1); 457 out of 6,554 proteins, 481 out of 6,478 proteins and 3,971 out of 10,316 proteins for COAD, HCC, and LUAD, respectively. The majority of those differentially abundant proteins were down-regulated in tumor samples (∼90% for COAD and HCC, 60% for LUAD) (Supplementary Figure S1A).

We next aimed to understand if complex subunits are to a lower degree affected by differential abundance changes when comparing tumors with healthy tissue. To do this, we performed an association test between the differentially abundant proteins, with up- and down-regulated proteins pooled, and protein complex subunits obtained from the CORUM database (Giurgiu et al., 2019). We observed a significant depletion of differentially abundant proteins in protein complex subunits for COAD and HCC (*p* < 0.0001, chi-square test) (Figure 1B). In order to avoid a potential bias due to the abundance differences between complex and non-complex proteins, we repeated the association test by using a size- and abundance-matched non-complex subunits as background. We could reproduce our previous result in COAD and HCC cohorts where we observed significant association. We hypothesize that complex subunits are protected from abundance changes as stoichiometric imbalances in protein complexes cause proteotoxicity and prevent proper functioning of complexes. Similar observations have been previously described for abundance changes triggered by focal genomic copy number alterations (Gonçalves et al., 2017).

For the LUAD cohort, we observed in relative terms a higher proportion of up-regulated proteins when compared to abundance changes in COAD and HCC, where around 90% of the differentially abundant proteins were down-regulated (Supplementary Figure S1A). A chi-square test was performed for up- and down-regulated proteins separately in the LUAD cohort where this comparison was feasible to assess differences in their overlap with the complex subunits. We observed that up-regulated proteins were significantly enriched in complex subunits (*p* = 6.28e-17, chi-square test) while a significant depletion was observed (consistent with the other cohorts) in the overlap between the down-regulated proteins and complex subunits (*p* = 8.42e-19, chi-square test) (Supplementary Figure S1B). The overall strong depletion of complex members among downregulated proteins suggests that complexes are protected from downregulation of their components as the lack of subunits will prevent the proper functioning of complexes, which might be more detrimental for the tumor than upregulation of components.

In summary, we observed that protein complex membership protects proteins from downregulation in cancer to a certain degree. Still, many protein abundance changes happen in cancer (Guang et al., 2019). Indeed, we observed a higher variation in protein abundances across tumor samples as compared to the matched normal samples in all of the 3 cohorts (*p* < 0.0001, *t*-test) (Figure 1C). We therefore decided to test if protein abundance correlation differs between complex partners vs. non-partners. To this end, we additionally included the CPTAC proteome data for the available TCGA projects (for which no matched normal samples were available and, hence, had been excluded from the differential abundance analysis): COREAD (including both colon and rectal tumors differently from the COAD cohort) (The Cancer Genome Atlas Network, 2012a; Zhang et al., 2014), OV (The Cancer Genome Atlas Network, 2011; Zhang et al., 2016) and BRCA (The Cancer Genome Atlas Network, 2012b; Mertins et al., 2016) cohorts. For all six cohorts (COAD, HCC, LUAD, COREAD, OV, and BRCA), we calculated protein level Spearman correlations for all possible pairs of proteins covered by CORUM complexes across tumor samples. We found that proteins involved in the same complex have significantly stronger protein abundance correlations (*p* < 2.2e-16, Wilcoxon test) (Figure 1D).

Together these observations suggest that protein complex organization constrains protein abundance changes in cancer, which is characterized by dysregulation of proteins, and has an impact on the strength of co-abundance patterns.

### Protein-Protein Interaction Classifications

To further understand how the dynamics of protein complex formation and the interaction between their components affect co-regulation of abundance changes of complex subunits, we first systematically categorized interaction types in complexes into five classes based on protein structure, proteomics measurements in different cellular conditions, biochemical properties of the binding interfaces, and literature information: i) stoichiometric ratio between proteins; ii) co-occurrence frequency of proteins; iii) context-specific vs. general interactions; iv) competitive vs. cooperative interactions; and v) transient vs. permanent interactions (Table 1). Next, we tested which of the complex interaction types contributes to the strength of abundance correlations between co-complex members in cancer. To do this, we calculated protein level Spearman correlations for all possible pairs of quantified proteins across tumor samples separately for each cohort included in this study (COAD, HCC, LUAD, COREAD, OV, and BRCA).

**TABLE 1:** Categories of PPIs. Five interaction types between proteins, their definition and the source from which the interaction information is obtained. The illustrations for each defined interaction type are in Figure 2.

Class	Definition	Source
Stoichiometric ratio between proteins	Copy numbers of proteins within a complex relative to each other	PDB Berman et al. (2000)
Co-occurrence frequency of proteins	The number of complexes two proteins are found together	CORUM database Giurgiu et al. (2019)
Context-specific vs. general interactions	PPIs are classified into two groups based on their detection in different human cell lines (HEK293T and HCT116)	BioPlex Interactome Huttlin et al. (2021)
Competitive vs. cooperative interactions	The terms, “competitive” and “cooperative,” are used to define the relationships between two proteins that bind to the same protein	Interactome Insider Meyer et al. (2018)
Transient vs. permanent interactions	Based on the stability of a complex and physicochemical characteristics of protein interfaces, protein-protein interaction can be grouped as transient, formed temporarily, and permanent interactions, naturally formed (Nooren and Thornton, 2003a; Nooren and Thornton, 2003b)	Block et al. (2006), Mintseris and Weng (2003)

**FIGURE 2:**
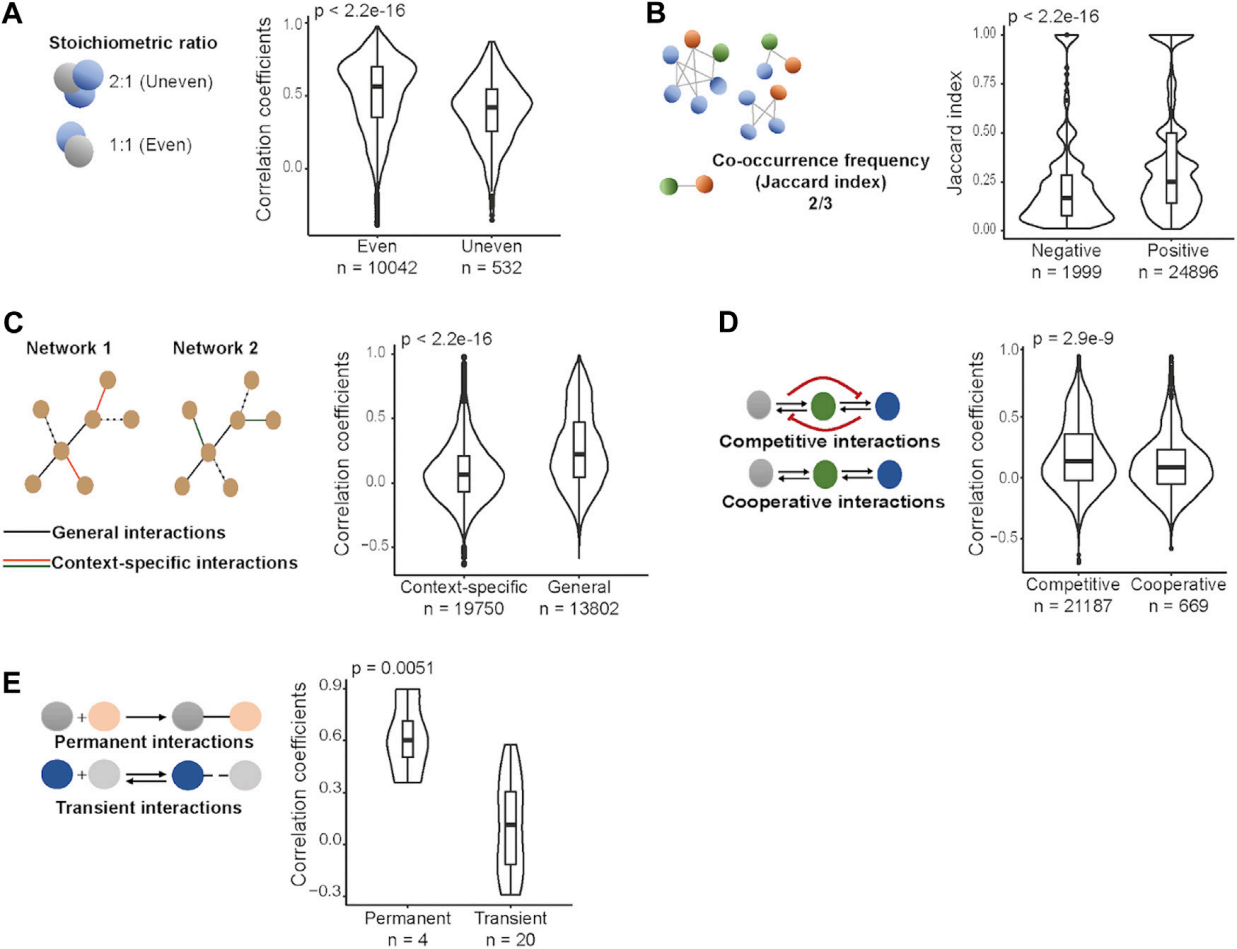
Protein-protein interaction types and protein level correlations between proteins across tumor samples in HCC. **(A)** Stoichiometric ratio between proteins. **(B)** Co-occurrence frequency of proteins. **(C)** Context-specific vs. general interactions. **(D)** Competitive vs. cooperative interactions. **(E)** Permanent vs. transient interactions. Wilcoxon test was performed to compare correlations between two groups in each class of PPIs.

Proteins interact with each other in varying stoichiometric ratios in protein complexes (Taggart et al., 2020). To test whether the stoichiometric ratio between co-complex members constrains abundance changes within a complex, we used structural data obtained from the PDB (Berman et al., 2000) for the available human heteromeric protein complexes (*n* = 8,388) and calculated the stoichiometric ratio between the proteins within the same complex. Then we grouped protein pairs as the ones with even stoichiometric ratio (e.g., 1:1, 2:2, 4:4) and those with uneven stoichiometric ratio (e.g., 2:1, 1:2, 3:1) and compared protein level correlations between these two groups in each cohort. We observed that protein pairs involved in complexes in an even stoichiometric ratio have significantly stronger correlations than other protein pairs (*p*-value < 0.0001, Wilcoxon test) in all cohorts (Figure 2A; Supplementary Figure S2A). We next tested if the stoichiometric ratio would have an impact on the steepness of the regression curve. We found, considering only the significant relationships (where coefficients are different from zero; *p*-value < 0.05, linear regression model), that positively correlated protein pairs with even stoichiometric ratio are associated with higher slopes (significant for 4 cohorts out of 6; *p*-value < 0.0001, Wilcoxon test) while their negatively correlated counterparts are associated with lower slopes (significant for 2 cohorts out of 6; *p*-value < 0.05, Wilcoxon test) (Supplementary Figure S3). These observations make sense as when a protein is upregulated its co-complex members that participate with a higher number of copies in the complex need to be upregulated to an even higher degree to fulfil the stoichiometric constraints.

Some protein pairs are found together in many complexes while others co-occur only in a few complexes. Thus, we asked if the frequency of the co-occurence of protein pairs in complexes has an effect on co-abundance changes in cancer. To do this, for each protein pair among CORUM protein complex subunits, we computed co-occurrence frequencies in complexes. Comparison of the co-occurrence frequencies between positively and negatively correlated proteins showed that positively correlated protein pairs tend to occur more often in the same complexes, consistently in each cohort (*p*-value < 0.0001, Wilcoxon test) (Figure 2B; Supplementary Figure S2B). This observation matches our expectation as frequent co-membership in complexes means a larger number of complexes will depend on the proper abundance ratios between the two proteins and this will increase the need for coregulation of the protein pair.

We further tested context-specific vs. general interactions. As a proxy for the interaction specificity, we included experimentally determined cell-line specific PPIs (Huttlin et al., 2021) and classified them as context-specific interactions if they were detected in only one of the two cell lines, and general interactions if present in both cell lines. We found that proteins interacting independently from the context are associated with stronger correlations than those interacting in a context-dependent manner in all cohorts (*p*-value < 0.0001, Wilcoxon test) (Figure 2C; Supplementary Figure S2C). The result makes sense as general interactions, by definition, are less prone to be affected by different cellular environments, and thus their co-abundance changes are expected to be more correlated.

While some proteins bind to few partners, some have multiple partners binding at similar (overlapping) interaction sites (Keskin and Nussinov, 2007). To assess whether competing for binding affects co-abundance changes in cancer, we first identified competitive and cooperative interactions, and then compared protein level correlations between them. To this end, we used experimentally determined human binary interactions curated from Interactome INSIDER (Meyer et al., 2018) and grouped them as competitive and cooperative interactions based on the similarity of their corresponding interaction sites on their shared partners. We observed that competitively interacting proteins have significantly higher correlations than cooperatively interacting proteins, consistently for 5 cohorts out of 6 (*p*-value < 0.05, Wilcoxon test) (Figure 2D; Supplementary Figure S2D). This can be robustly reproduced when comparative and cooperative interactions were grouped based on different binding similarity scores (Supplementary Figure S4). This observation surprised us as we expected weaker or negative correlations between competitively interacting proteins as those, by definition, should not participate in the same complex at the same time and hence an opposing expression pattern would be expected.

Based on stability, PPIs are classified as permanent and transient interactions. We obtained permanent and transient interactions estimated by machine learning algorithms based on physicochemical properties of PPIs from Minteris and Weng (2003) and Block et al. (2006), and then compared protein level correlations between those two groups. While not significant in each single cohort, we observed consistent trends: permanent interactions correspond to stronger correlations while relatively weaker, transient interactions were observed between proteins whose abundances are less dependent on each other, and this trend is statistically significant in COREAD, COAD, and HCC cohorts (*p*-value < 0.05, Wilcoxon test) (Figure 2E; Supplementary Figure S2E). This is expected as the transient interactions are more flexible for a change in binding partners during the assembly of complexes (Nooren and Thornton, 2003a).

## Discussion

Cancer is characterized by many alterations including transcriptome and proteome dysregulation. There seems to be a larger number of dysregulated transcripts as compared to proteins (Stingele et al., 2012). This suggests compensatory mechanisms on translation level and raises the interesting question which of the changes in gene expression are tolerated by the cancer cell and which not. Our analysis reconfirms that complex members are to a certain degree protected from abundance changes that could mess with complex stoichiometry. In addition, we showed that the type of interaction within a protein complex constrains proteome abundance and dysregulation to maintain functional complex organization. To address this, we systematically categorized interaction types in complexes by integrating experimental measurements and computational predictions, and tested each category for its impact on abundance changes by using cancer proteomics data.

In most yet not in every case, our observations matched our expectations. However, in some comparisons we observed different degrees of the expected trend in different cohorts: e.g., for the observation that permanent interactions are associated with higher correlations (Figure 2E; Supplementary Figure S2E). This variation might be explained by the way the physiological conditions and local environment affect the stability of an interaction meaning that permanent interaction may become transient under certain conditions or vice versa (Nooren and Thornton, 2003a). Additionally, computational prediction of transient and permanent interactions might not fully capture how proteins interact in different local environments represented by different cancer types used in this study.

For proteins binding to the same proteins through overlapping interaction sites, competition is expected. We hypothesized that proteins competing with each other to bind their common partners will show weaker correlations in their protein abundances. However, we were surprised that the results were in the opposite direction of our expectation (Figure 2D; Supplementary Figure S2D). Those results could be reproduced over a range of thresholds for binding site overlap definition (Supplementary Figure S4). The reason could be that alternative regulatory mechanisms prevent competition between partners (Li et al., 2015) or that our computational estimates of competition vs. cooperative binding are not accurate. For example, biophysical properties of interaction sites of proteins (e.g., steric hindrance) can favor or prevent interactions between them and thus, the classification of proteins based on the overlap in the interaction interface might not represent competitive or cooperative binding in reality. In addition, it should be noted that those estimates are based on the measurement from *in vitro* experiments. The proteins classified as competing could have different localization or be expressed in different cells/tissues *in vivo* and in reality never meet.

The purpose of this study was to provide a proof of concept that interaction types within complexes affect co-abundance patterns in cancer. To this end, we picked only a limited set of studies to classify interactions or quantify protein abundances. Hence, the primary limitation of our study is the small number of representative examples for several types of interactions such as permanent vs. transient interactions where mining the literature revealed only a few instances. Additionally, for the class of context-specific vs. general interactions, we considered the proteomics measurements of two different cell lines. Thus, integrating a larger number of cell-line specific proteomics interaction datasets will potentially provide a more comprehensive understanding in the future.

## Data Availability

The datasets analyzed in this study are publicly available and can be found here: Proteomics data in the CPTAC (https://cptac-data-portal.georgetown.edu/), the known human protein complexes in the CORUM database (http://mips.helmholtz-muenchen.de/corum/#download), structural information of protein complexes in the PDB (rcsb.org), 121,575 experimentally determined human binary interactions in Interactome INSIDER (http://interactomeinsider.yulab.org/downloads.html), and cell-line specific PPIs in BioPlex Interactome (https://bioplex.hms.harvard.edu/interactions.php). The datasets generated for this study can be found as part of PPIs_Data-Code repository at (https://github.com/SengerG/PPIs_Data-Code).
