# Supplementary material for: Protein Complex Organization Imposes Constraints on Proteome Dysregulation in Cancer
Source: Front Bioinform. 2021 Aug 30;1:723482. doi: 10.3389/fbinf.2021.723482 (PMC9580999; doi:10.3389/fbinf.2021.723482)
Supplement: Supplementary file 1 [file DataSheet1.pdf]

Supplementary Material

Supplementary Table 1. Number of proteins with different stoichiometric ratios and their classification as even and uneven in each cohort.

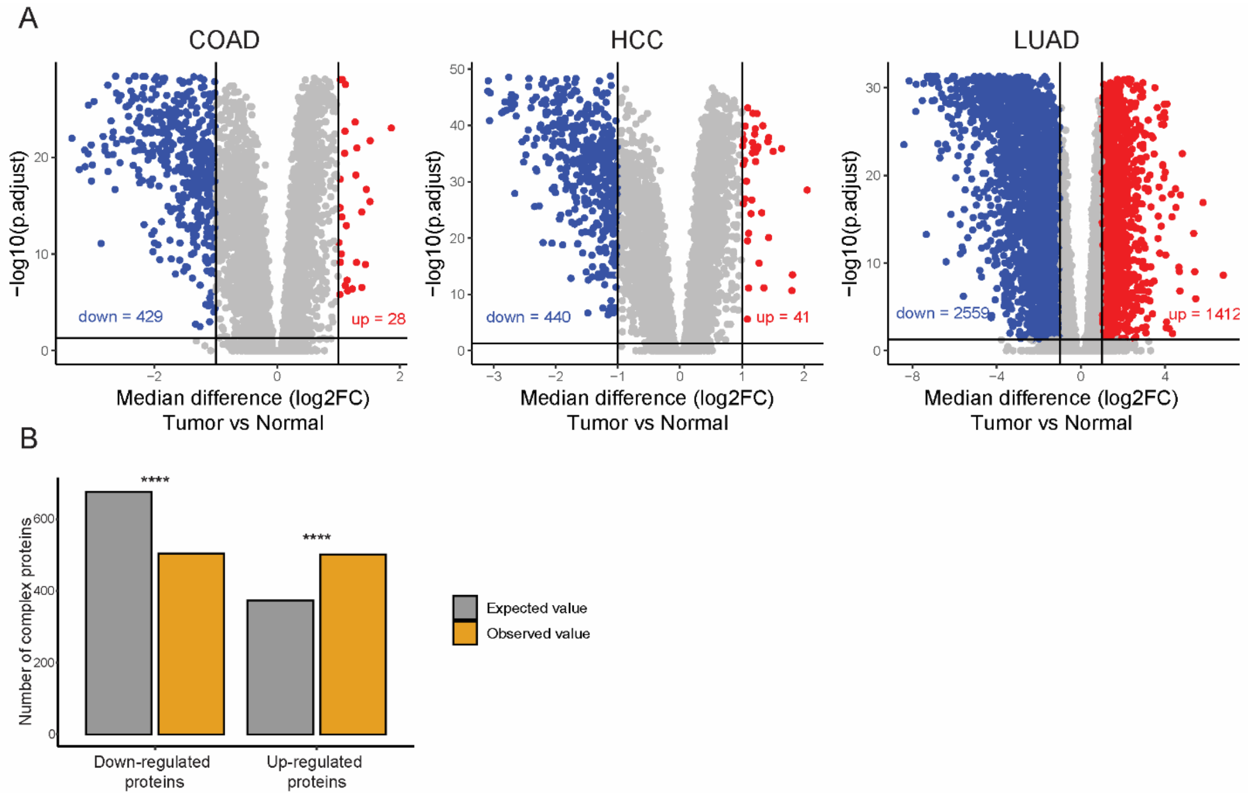

Supplementary Figure 1. Protein abundance changes in cancer. (A) Differential protein abundance changes in COAD, HCC and LUAD cohorts. **(B)** The expected and observed number of complex subunits in up- and down-regulated proteins in LUAD cancer. The chi-square test was used to determine if the difference between expected and observed values is statistically significant (****: P < 0.0001).

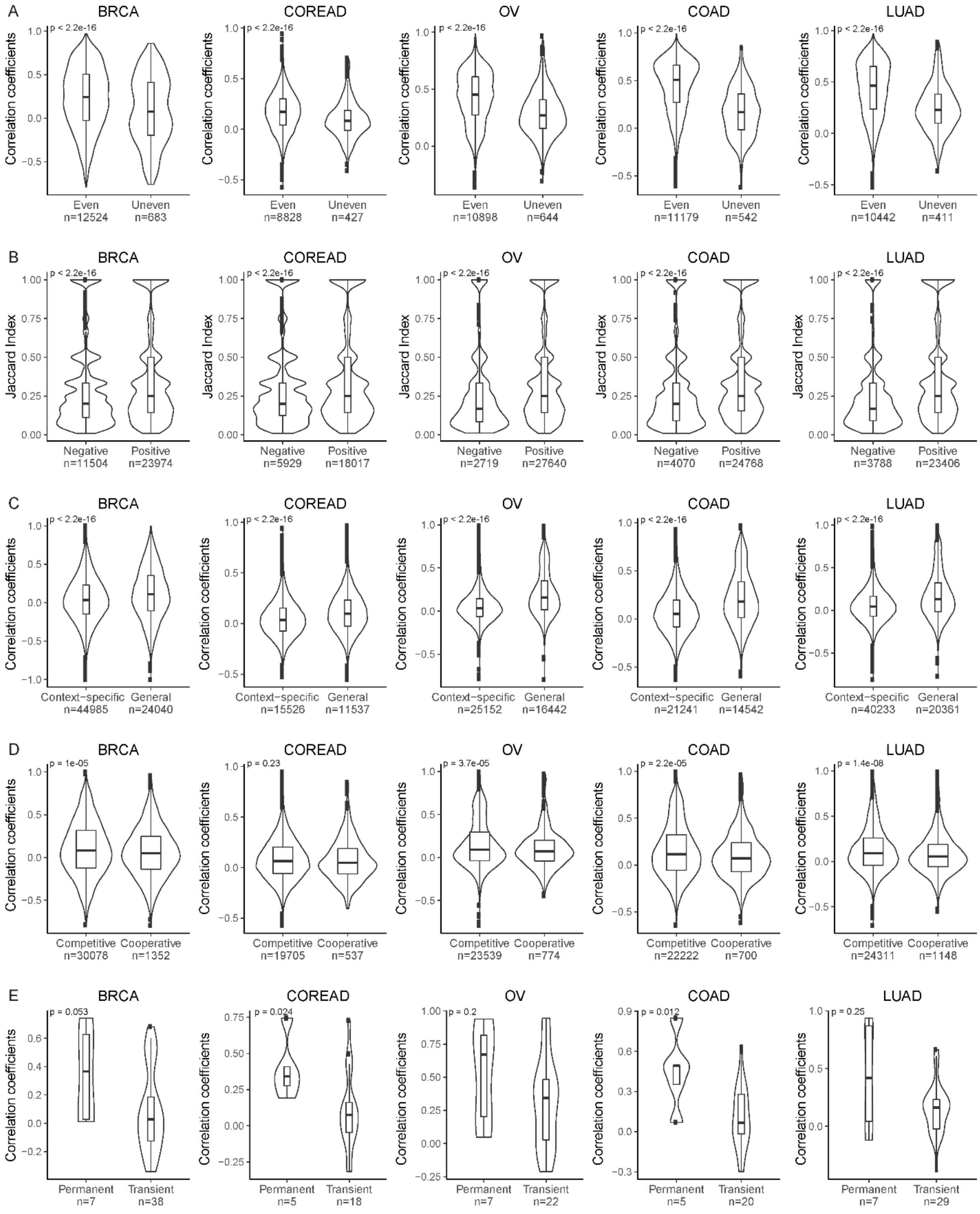
A
BRCA
COREAD
OV
COAD
LUAD
Correlation coefficients
p < 2.2e-16
Even n=12524
Uneven n=683
Even n=8828
Uneven n=427
Even n=10898
Uneven n=644
Even n=11179
Uneven n=542
Even n=10442
Uneven n=411
B
BRCA
COREAD
OV
COAD
LUAD
Jaccard Index
p < 2.2e-16
Negative n=11504
Positive n=23974
Negative n=5929
Positive n=18017
Negative n=2719
Positive n=27640
Negative n=4070
Positive n=24768
Negative n=3788
Positive n=23406
C
BRCA
COREAD
OV
COAD
LUAD
Correlation coefficients
p < 2.2e-16
Context−specific n=44985
General n=24040
Context−specific n=15526
General n=11537
Context−specific n=25152
General n=16442
Context−specific n=21241
General n=14542
Context−specific n=40233
General n=20361
D
BRCA
COREAD
OV
COAD
LUAD
Correlation coefficients
p = 1e-05
p = 0.23
p = 3.7e-05
p = 2.2e-05
p = 1.4e-08
Competitive n=30078
Cooperative n=1352
Competitive n=19705
Cooperative n=537
Competitive n=23539
Cooperative n=774
Competitive n=22222
Cooperative n=700
Competitive n=24311
Cooperative n=1148
E
BRCA
COREAD
OV
COAD
LUAD
Correlation coefficients
p = 0.053
p = 0.024
p = 0.2
p = 0.012
p = 0.25
Permanent n=7
Transient n=38
Permanent n=5
Transient n=18
Permanent n=7
Transient n=22
Permanent n=5
Transient n=20
Permanent n=7
Transient n=29

Supplementary Figure 2. Protein-protein interaction types and protein level correlations between proteins across tumor samples for five cohorts (BRCA, COREAD, OV, COAD, LUAD). (A) Stoichiometric ratio between proteins. **(B)** Co-occurrence frequency of proteins. **(C)** Context-specific vs general interactions. **(D)** Competitive vs cooperative interactions. **(E)** Permanent vs transient interactions. Wilcoxon test was used to compare correlations between different protein interaction types.

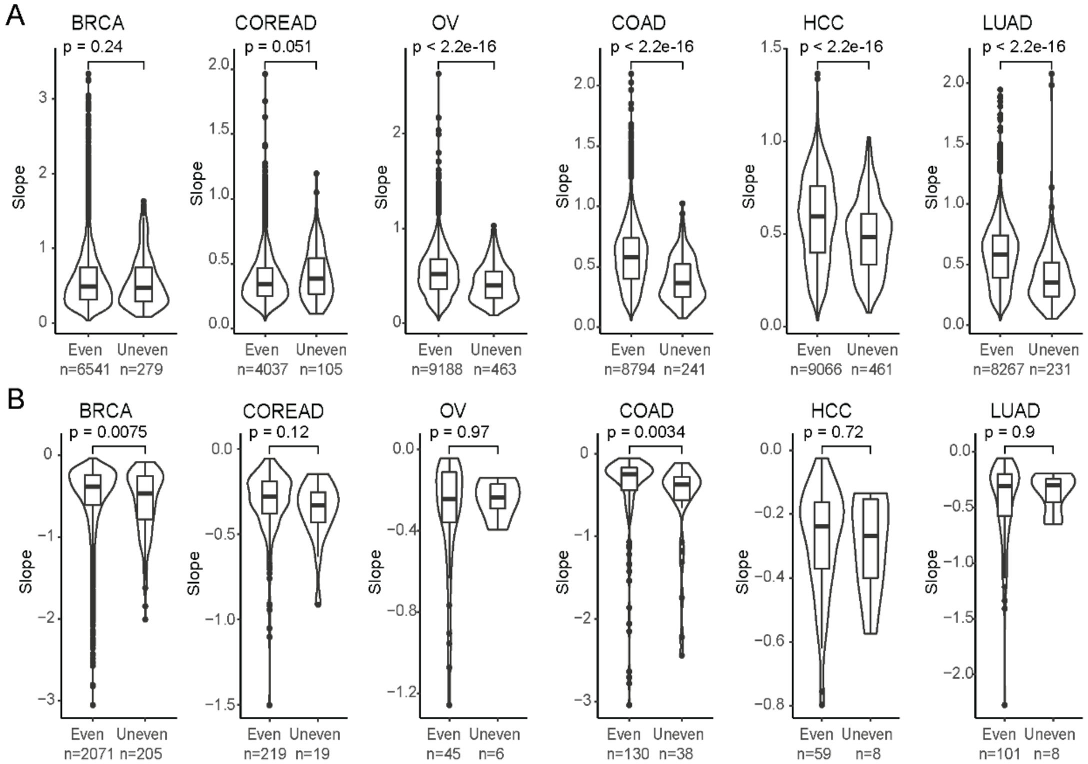

Supplementary Figure 3. Linear regression analysis for protein abundances. Comparison of distributions of slopes from the linear regression analysis between protein pairs with even and uneven stoichiometric ratios for each cohort (BRCA, COREAD, OV, COAD, HCC, LUAD). **(A)** Positively correlated proteins and **(B)** negatively correlated proteins. After regression analysis, only the significant results ($p < 0.05$, linear regression model) were considered.

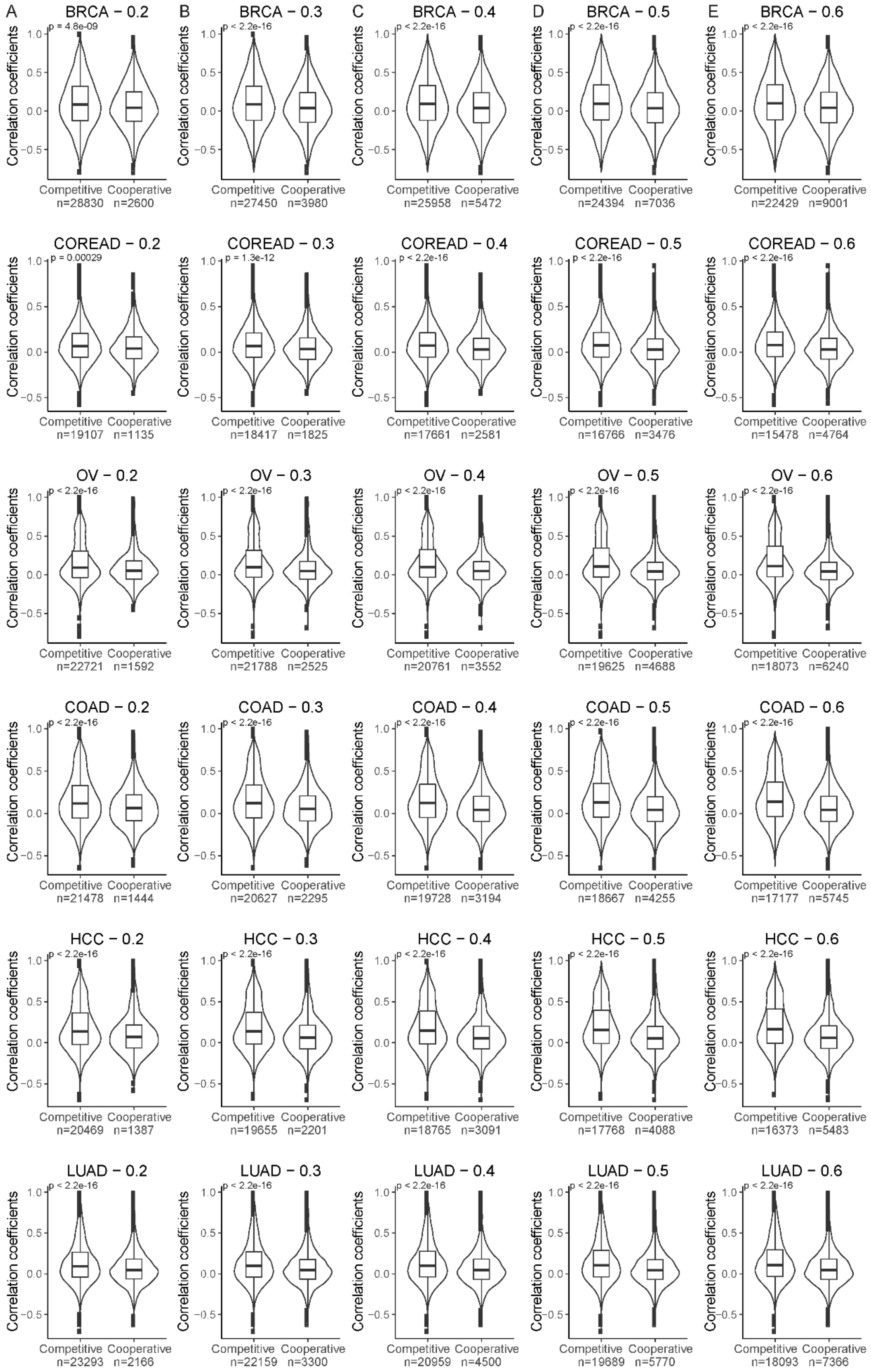
A
BRCA − 0.2
p = 4.8e-09
Competitive n=28830
Cooperative n=2600
B
BRCA − 0.3
p < 2.2e-16
Competitive n=27450
Cooperative n=3980
C
BRCA − 0.4
p < 2.2e-16
Competitive n=25958
Cooperative n=5472
D
BRCA − 0.5
p < 2.2e-16
Competitive n=24394
Cooperative n=7036
E
BRCA − 0.6
p < 2.2e-16
Competitive n=22429
Cooperative n=9001
COREAD − 0.2
p = 0.00029
Competitive n=19107
Cooperative n=1135
COREAD − 0.3
p = 1.3e-12
Competitive n=18417
Cooperative n=1825
COREAD − 0.4
p < 2.2e-16
Competitive n=17661
Cooperative n=2581
COREAD − 0.5
p < 2.2e-16
Competitive n=16766
Cooperative n=3476
COREAD − 0.6
p < 2.2e-16
Competitive n=15478
Cooperative n=4764
OV − 0.2
p < 2.2e-16
Competitive n=22721
Cooperative n=1592
OV − 0.3
p < 2.2e-16
Competitive n=21788
Cooperative n=2525
OV − 0.4
p < 2.2e-16
Competitive n=20761
Cooperative n=3552
OV − 0.5
p < 2.2e-16
Competitive n=19625
Cooperative n=4688
OV − 0.6
p < 2.2e-16
Competitive n=18073
Cooperative n=6240
COAD − 0.2
p < 2.2e-16
Competitive n=21478
Cooperative n=1444
COAD − 0.3
p < 2.2e-16
Competitive n=20627
Cooperative n=2295
COAD − 0.4
p < 2.2e-16
Competitive n=19728
Cooperative n=3194
COAD − 0.5
p < 2.2e-16
Competitive n=18667
Cooperative n=4255
COAD − 0.6
p < 2.2e-16
Competitive n=17177
Cooperative n=5745
HCC − 0.2
p < 2.2e-16
Competitive n=20469
Cooperative n=1387
HCC − 0.3
p < 2.2e-16
Competitive n=19655
Cooperative n=2201
HCC − 0.4
p < 2.2e-16
Competitive n=18765
Cooperative n=3091
HCC − 0.5
p < 2.2e-16
Competitive n=17768
Cooperative n=4088
HCC − 0.6
p < 2.2e-16
Competitive n=16373
Cooperative n=5483
LUAD − 0.2
p < 2.2e-16
Competitive n=23293
Cooperative n=2166
LUAD − 0.3
p < 2.2e-16
Competitive n=22159
Cooperative n=3300
LUAD − 0.4
p < 2.2e-16
Competitive n=20959
Cooperative n=4500
LUAD − 0.5
p < 2.2e-16
Competitive n=19689
Cooperative n=5770
LUAD − 0.6
p < 2.2e-16
Competitive n=18093
Cooperative n=7366
Correlation coefficients
1.0
0.5
0.0
−0.5

Supplementary Figure 4. Comparison of protein abundance correlations between competitive and cooperative interactions when they were grouped based on different binding similarity score cut-offs. Each panel shows the comparison between groups categorized based on a certain cut-off for all cohorts (BRCA, COREAD, OV, COAD, HCC, LUAD). The interaction between two proteins is competitive, if the binding similarity score is equal to or larger than **(A)** 0.2, **(B)** 0.3, **(C)** 0.4, **(D)** 0.5, and **(E)** 0.6, otherwise cooperative.
